# Impact of increased diagnosis of early HIV infection and immediate antiretroviral treatment initiation on HIV transmission among men who have sex with men in the Netherlands

**DOI:** 10.1371/journal.pcbi.1012055

**Published:** 2025-02-27

**Authors:** Alexandra Teslya, Janneke Cornelia Maria Heijne, Maarten Franciscus Schim van der Loeff, Ard van Sighem, Jacob Aiden Roberts, Maartje Dijkstra, Godelieve J de Bree, Axel Jeremias Schmidt, Kai J Jonas, Mirjam E Kretzschmar, Ganna Rozhnova

**Affiliations:** 1 Julius Center for Health Sciences and Primary Care, University Medical Center Utrecht, Utrecht University, Utrecht, The Netherlands; 2 Department of Infectious Diseases, Public Health Service of Amsterdam, Amsterdam, The Netherlands; 3 Amsterdam Institute for Immunology & Infectious Diseases (AII), Amsterdam UMC, University of Amsterdam, Amsterdam, The Netherlands; 4 Amsterdam Public Health Research Institute (APH), Amsterdam UMC, University of Amsterdam, Amsterdam, The Netherlands; 5 Department of Internal Medicine, Division of Infectious Diseases, Amsterdam Institute for Immunology and Infectious Diseases, Amsterdam UMC, University of Amsterdam, Amsterdam, The Netherlands; 6 Stichting HIV Monitoring, Amsterdam, The Netherlands; 7 Amsterdam UMC location University of Amsterdam, Amsterdam Public Health Research Institute, Amsterdam, The Netherlands; 8 Sigma Research, Department of Public Health, Environments and Society, London School of Hygiene and Tropical Medicine, London, United Kingdom; 9 Medicine and Health Policy Unit, German AIDS Federation, Berlin, Germany; 10 Faculty of Psychology and Neuroscience, Maastricht University, Maastricht, The Netherlands; 11 Center for Complex Systems Studies (CCSS), Utrecht University, Utrecht, The Netherlands; 12 Institute of Epidemiology and Social Medicine, University of Münster, Münster, Germany; 13 BioISI – Biosystems & Integrative Sciences Institute, Faculdade de Ciências, Universidade de Lisboa, Lisbon, Portugal; 14 Faculdade de Ciências, Universidade de Lisboa, Lisbon, Portugal; University of Zurich, SWITZERLAND

## Abstract

The number of new HIV infections among men who have sex with men (MSM) in the Netherlands has been decreasing, but additional efforts are required to bring it further down. This study aims to assess the impact of increased diagnosis of early HIV infection combined with immediate antiretroviral treatment (ART) initiation on reducing HIV transmission among MSM. We developed an agent-based model calibrated to HIV surveillance and sexual behavior data for MSM in the Netherlands in 2017-2022. Starting in 2023, we simulated a 10-year intervention that accelerates HIV diagnosis during the first 3 or 6 months after HIV acquisition across five levels of increased diagnosis rates (2, 4, 8, 16, and 32-fold), followed by immediate ART initiation. The upper limit of the intervention’s impact over 10 years is projected to result in the cumulative 298 (95-th QI: 162–451) HIV infections averted. A 32-fold increase in the diagnosis rate within 3 months after HIV acquisition (corresponding to 100% of all new HIV infections diagnosed within 3 months of acquisition) results in 269 (95-th QI: 147–400) infections averted, approaching closely maximum impact. By extending the scope of the intervention to individuals who acquired HIV infection within the previous 6 months, a smaller 8-fold increase in the diagnosis rate (corresponding to 97% of new HIV infections diagnosed within 6 months of acquisition) approaches closely the maximum impact of the intervention by averting 256 (95-th QI: 122–411) HIV infections. Our sensitivity analyses showed that, in an epidemiological context similar to the modern-day the Netherlands, immediate initiation of ART accompanying accelerated diagnosis of individuals with early HIV infection does not significantly affect HIV transmission dynamics. Accelerating early HIV diagnosis through increased awareness, screening, and testing can further reduce transmission among MSM. Meeting this goal necessitates a stakeholder needs assessment.

## Author summary

In recent years, in the Netherlands, the annual number of new HIV infections in the population of men who have sex with men (MSM) is declining. Using an agent-based model calibrated to the current state of HIV epidemic in MSM in the Netherlands, we explored the potential impact of an intervention that accelerates diagnosis in individuals with early HIV infection and facilitates immediate antiretroviral therapy (ART) initiation to reduce the number of new HIV infections in the population of MSM even further. Our projections indicate that such an intervention can noticeably reduce onward HIV transmission in the population of MSM. Increase in diagnosis rate by a factor of 32 for individuals with early HIV infection results in the impact close to the maximum (simulated by modeling newly infected individuals instantaneously starting ART), but still leaves the room for improvement. Extending the intervention to target individuals who acquired HIV infection within the previous 6 months could by a factor 8 could result in similar number of HIV infections averted. Immediate initiation of ART following the diagnosis while key in order to prevent deterioration of CD4 cell count, does not have significant effect on the HIV transmission dynamics. The intervention which achieves increased diagnosis rate in individuals with early HIV who then immediately initiate ART, can bring forth substantial reductions, even against a backdrop of an already declining trend.

## Introduction

In recent years, the Netherlands has made significant progress in containing the HIV epidemic among men who have sex with men (MSM). This success is evident in observable epidemic trends, such as the decreasing numbers of annual new HIV diagnoses [1] and can be attributed to effective care and prevention programs aligning closely with the 95-95-95 UNAIDS goals set for 2025 [2]. The goals define the percentages of diagnosed individuals out of the total population with HIV, individuals using antiretroviral treatment (ART) out of the total diagnosed population, and individuals with suppressed viral load out of the total population on ART, collectively referred to as the cascade of care. While the UNAIDS 95-95-95 goals currently stand at 96-97-97 for the overall MSM population in the Netherlands [1], HIV transmission is still ongoing within this group [3].

In 2022, MSM remained one of the key populations, accounting for more than 54% of new HIV diagnoses in the Netherlands [1]. The estimated annual number of newly acquired HIV infections among MSM has been steadily decreasing from 660 (CI: 620–720) in 2010 to 80 (CI: 40–150) in 2022 [1]. To reach the goal of zero new HIV infections, intervention efforts must be scaled up, and additional measures might be required to further reduce onward transmission. On one hand, addressing late diagnosis and care initiation remains an important goal. To wit, in 2022, a substantial proportion of new HIV diagnoses in MSM occurred in late and advanced stages of HIV infection, with 34% and 21% of all diagnoses, respectively [1]. On the other hand, Ratmann et al. [4], analyzing data collected from 1996 to 2010, estimated that 43% of recent HIV infections (within 1 year) were linked to exposure from individuals who had themselves acquired HIV infection within the preceding 12 months. Thus, increasing the diagnosis rate of MSM with early HIV infection and swiftly linking them to care to achieve an undetectable viral load is crucial. This strategy is paramount for achieving HIV elimination. Importantly, it will also prevent the decline of CD4 cell counts and subsequently halt the progression of health deterioration in MSM with HIV.

In the early 2000s, ART initiation was guided by the declining count of CD4 cells in people with HIV. In 2015, in response to the INSIGHT trial results [5], a new recommendation advocating for early treatment initiation, regardless of CD4 count, was adopted. The study by Kroon et al. [6] estimated that immediate ART initiation by individuals who were diagnosed in the acute stage of HIV infection could reduce the projected number of new HIV infections by approximately a factor of 5, compared to the scenario without early detection and immediate ART initiation. A systematic review and meta-analysis by Ford et al. [7] concluded that ART initiation on the day of diagnosis increases the odds of achieving suppression and remaining in care after 1 year. Zhao et al. [8] observed that a decrease in the time between HIV diagnosis and ART initiation was associated with increased odds of achieving viral suppression within 1 year of initiation. Thus, decreasing the time between diagnosis and ART initiation could contribute to a reduction in onward transmission.

Targeted screening for early HIV infection and immediate ART initiation among MSM was demonstrated to be a feasible and effective strategy at the Amsterdam sexually transmitted infection clinic [9]. The strategy involved a media campaign aiming to increase awareness of early HIV infection among MSM [10], a rapid diagnostic test for early infection, and a same-day referral trajectory to initiate ART. In this strategy, MSM with possible early infection, identified using a validated early-infection risk score [11], received point-of-care HIV-RNA testing. MSM diagnosed with early infection were immediately referred to start ART within 24 hours. This approach resulted in increased early HIV infection diagnoses and reduced the time from diagnosis to viral suppression [9]. A national rollout of such a strategy could move us closer to eliminating the HIV epidemic among MSM in the Netherlands. However, for the effective implementation of this intervention at the population level, it is essential to determine the optimal parameters, including the specific increase needed in the diagnosis rate and the precise target populations. While investigating the population-level effects of this strategy empirically is challenging, mathematical models can be used to determine its potential as a public health intervention.

Currently, there are no modeling studies evaluating the impact of an intervention aiming to accelerate the diagnosis of MSM with early HIV infection combined with immediate ART in the Netherlands. Building on the findings of the studies outlined above, we investigate the effectiveness of such an intervention in the Dutch setting. Our study aims to provide novel insights that could enhance the development of interventions aimed at advancing the elimination of HIV, particularly in settings with low rates of new HIV infections.

## Materials and methods

### Model overview

To project HIV transmission dynamics among MSM in the Netherlands, the developed agent-based model captures three types of processes: demographic dynamics, sexual network dynamics, and HIV disease progression and transmission dynamics. The demographic dynamics include the entrance of new individuals into the population, aging, and the exit of individuals from the population due to background mortality or cessation of sexual activity. The sexual network dynamics capture steady (long-term, non-overlapping with other steady partnerships) and non-steady (short-term, with the potential for concurrency) sexual partnerships that form and dissolve dynamically. HIV disease progression and transmission capture the natural history of HIV infection, and current practices of HIV care and prevention in the Netherlands, such as the cascade of care and pre-exposure prophylaxis (PrEP). The model accounts for importation of HIV infections from outside of the Netherlands, either as a result of residents of the Netherlands acquiring HIV while traveling abroad or due to individuals immigrating to the Netherlands with an existing infection. The simulation advances one day at a time.

### Demography

The modeled population consisted of 25,000 individuals, scaled down from an estimated MSM population in the Netherlands of 200,000–300,000 individuals [12]. We considered sexually active individuals between 15 and 75 years of age. Individuals enter the population at the age of 15 years, age as the simulation advances, and exit the population once they reach 75 years. They can also leave the population as a result of background age-dependent mortality.

### Sexual network dynamics

In the context of MSM, anal intercourse (AI) is the predominant mode of transmission [13]. We model HIV transmission through AI in steady and non-steady sexual partnerships. Individuals can enter new partnerships, and existing partnerships can dissolve. A steady partnership is a long-term partnership. Individuals can have at most one steady partner at a time. All individuals in the population have the same propensity to enter a steady partnership. The term non-steady partnership designates all other partnerships where AI can take place. Non-steady partnerships are short-term and may be concurrent with other partnerships, both steady and non-steady. The propensity to enter new non-steady partnerships is an intrinsic property of an individual and depends on age and on being in a steady partnership. This property may change as individuals age, or when their steady-partner state changes. The formation of both types of partnerships is influenced by the effects of age and HIV status on selecting a partner, referred to as age assortativity and serosorting, respectively.

### HIV dynamics

We use a standard HIV framework for modeling the natural history of HIV infection, which is viewed as the sequence of early, chronic, and AIDS infection stages. In our study, we adopt an approach similar to that by Dijkstra et al. [9], where early infection lasts, on average, 89 days. This duration aligns with the cumulative duration of the first five stages of infection in the Fiebig classification [14] that delineates substages of early infection corresponding to distinct levels of HIV viremia, antibody seroconversion responses, and sensitivity of diagnostic methods needed to detect HIV infection. After an infection has occurred, individuals who acquired HIV progress through the early infection stage. Subsequently, individuals enter the chronic infection stage, which is an extended period where the potential to transmit HIV remains approximately constant and significantly lower than in the early infection stage. Once the chronic infection stage has concluded, individuals enter the AIDS stage, which we further divide into early and late AIDS stages. The early AIDS stage is characterized by an increased probability of transmitting HIV via AI compared to the chronic stage and additional mortality due to AIDS. Finally, a rare occurrence in the Netherlands, individuals who survive the early AIDS stage enter the late AIDS stage. This stage is characterized by a rapid rise in viral load, cessation of sexual activity, and increased mortality relative to the early AIDS stage.

### Baseline care and prevention

The model captures the current routine HIV care and prevention programs in the Netherlands, including diagnosis-treatment-suppression cascade and PrEP. Since September 2019, PrEP can be obtained through the national PrEP program and via a prescription from general practitioners. We model continuous PrEP use, resulting in a substantial reduction in the likelihood of HIV acquisition during anal intercourse with individuals with HIV. We approximate the eligibility criteria for PrEP by considering the number of sexual partners in the past 6 months. The rates of enrollment in and exit from the PrEP program are calibrated so that, following the initial years of the PrEP rollout, 5% of the population is using PrEP. Individuals who acquire HIV may be diagnosed, subsequently initiate ART, and eventually achieve viral suppression, rendering them effectively incapable of transmitting HIV. A fraction of individuals may discontinue ART or experience ART failure, returning to a state with an unsuppressed viral load. For simplicity, we assume that these individuals can be offered ART again. We do not explicitly simulate testing or account for differences in testing rates across various individual characteristics. Instead, we model diagnosis rates, which are dependent on the stage of infection.

### Importation of HIV infections from outside of the Netherlands

To capture importation of HIV infections arising outside of the transmission network within the Netherlands, we assign a positive HIV status to a proportion of individuals who enter the population. Individuals with HIV can enter the population either aware or unaware of their status. The rate of entrance of individuals who know their HIV status is extrapolated using the Stichting HIV monitoring (SHM) reported data for years 2017-2022 [1,15–20], according to which about approximately 95% of incoming individuals who know their HIV status, have initiated ART treatment, and have a suppressed viral load. We model that these individuals continue ART treatment in the Netherlands. The annual number of individuals who either acquire HIV infection abroad or immigrate to the Netherlands with existing HIV infection and do not know their status cannot be estimated directly from the data, and thus we conflated these two sources of new HIV infections arising from outside of the Netherlands into a single parameter. The distribution for this parameter was estimated through the calibration process.

### HIV transmission within partnerships

HIV transmission between two sexual partners via AI depends on the HIV infection stage of the partner with HIV, the effective sexual contact rate between the two partners, and the use of PrEP. The effective sexual contact rate can be further broken down into the frequency of AI acts and the probability of condom use during these acts. Condom use depends on the type of partnership and on PrEP use by individuals in the partnership and results from reconciling the personal, age-dependent preferences of individuals. We model condom use as constant for the entire duration of the partnership. The frequency of AI acts differs per type of partnership, with everyone modeled as having the same preference for the frequency of AI. Finally, based on existing evidence [21], we model that the effective sexual contact rates in serodiscordant partnerships where the partner with HIV is diagnosed are reduced compared to the baseline level of partnerships with unknown HIV status. We assumed the reduction to be by a factor of 2.

### Model parametrization and calibration

To accurately project HIV transmission dynamics in the MSM population in the Netherlands, we use available data to set model parameter values. Most parameter values are determined through direct calculation, known as parametrization, whereas others are derived by adjusting the model outputs to fit available data, a method called calibration. This approach is used for parameters that are particularly difficult to measure directly, such as the probability of transmission by an individual with chronic HIV infection during condomless AI.We estimate diagnosis rates of individuals at various stages of HIV infection, by calibrating the model to the annual distribution of infection stages at time of diagnosis, reported by SHM. In this way we ensure that the model accurately reproduces the pattern of diagnosis rates in MSM in the Netherlands. To set parameters relevant to sexual network dynamics and sexual behavior within partnerships, either through the direct calculation from data or via the calibration process, we use data collected from several sexual network/behavior surveys among MSM in the Netherlands, i.e., the cross-sectional European MSM Internet Survey 2017 (EMIS-2017) [22], the longitudinal Amsterdam Cohort Studies (ACS) [23], and the cross-sectional Amsterdam MSM Network study [24,25]. To estimate HIV transmission dynamics, we use HIV surveillance data for MSM in the Netherlands [1,15–20,26] and estimates from the literature [27–29]. Employing parametrization and calibration procedures, we obtain the 100 parameter sets for which the distance between the median of the model simulation and the HIV surveillance estimate/data [1] was minimized and which were used to produce the analyses. For the selected parameter sets, the simulation median always falls within the estimate of the confidence interval for the data. For a comprehensive description of parameter estimation, consult the S1 Appendix.

#### Parametrization

**Demographic processes.** We used data from Statistics Netherlands to calculate background age-dependent mortality rates and the age distribution of the MSM population. The latter was assumed to be similar to the age distribution of the general male population [30]. The entrance rate of new individuals is a Poisson-distributed process with its mean set such that the population size fluctuates around 25,000 individuals.

**Sexual network.** Using available data, we calculated the proportion of the population that has a steady partner and the rates of acquisition of non-steady partners distributed by the age of individuals and the existence of a steady partner.

**HIV transmission dynamics.** Transition rates between HIV infection stages and the relative infectivity per contact in different HIV stages were parameterized using estimates from the literature [27–29].

#### Calibration

**Sexual network.** To obtain the rates of partnership formation and dissolution, we calibrated the model against the distribution of the rate of change of steady partners in the previous 12 months and the rate of change of non-steady partners in the previous 6 months using the data collected in EMIS-2017 [22] survey. To determine assortativity mixing and serosorting patterns, we calibrated the model against data collected in the Amsterdam MSM Network study [24,25] and Amsterdam Cohort Studies [23].

**HIV transmission dynamics.** To estimate the baseline probability of HIV transmission per condomless AI, the annual importation rate of new HIV infections, the diagnosis rates of individuals in different HIV stages, ART uptake, and viral suppression rates, we calibrated HIV transmission dynamics in the presence of currently realized standards of HIV care and prevention. The calibration targets were the estimated annual number of newly acquired HIV infections (calculated using HIV Platform Tool [31] and provided by SHM), the annual number of newly diagnosed HIV infections, the proportion of individuals diagnosed within specific time window after HIV acquisition (first 6 months, 6 to 12 months, and after more than 12 months) in the MSM population for the years 2017-2022 in the Netherlands [1,16–20].

### Model scenarios

In all scenarios, at the beginning of the simulation the population age structure is seeded using the state of the male population in 2014. We initiate HIV dynamics using the population distribution in the cascade of care in 2016 [15] and allow it to progress until 2023. Depending on the modeled scenario, an intervention is then initiated and run for 10 years starting in 2023. We simulate the following scenarios:

1. **Baseline scenario.** No additional interventions are modeled, except for the PrEP program and the cascade of care using the standard of care in 2016, wherein ART initiation increasingly occurred soon after HIV diagnosis.
2. **Intervention scenario.** An intervention that facilitates an increased diagnosis for individuals with early HIV infection and immediate ART initiation would start in 2023. We considered scenarios with the following specifications:
  - **Maximum impact scenario.** In this scenario, we simulate that every individual, whether with newly acquired HIV infection or with HIV infection acquired within the previous 6 months, begins ART right at the point of HIV acquisition or at the start of the intervention, whichever comes first. We achieve this by setting the diagnosis rates high enough to ensure immediate ART initiation for all eligible individuals upon HIV acquisition or with the intervention’s initiation. Although this scenario may not be realistic, examining its outcomes helps us understand the maximum potential impact of the intervention which combines increased diagnosis in individuals with early HIV infection with immediate initiation of ART.
  - **Accelerated diagnosis in individuals with early HIV infection.** In this scenario, we explore incremental increases in the diagnosis rate for individuals with early HIV infection, combined with immediate initiation of ART. Ultimately, we aim to identify the sufficient increase rate of the diagnosis rate in order to approach the maximum impact of the intervention. The diagnosis rates are increased relative to the baseline level by factors of 2, 4, 8, 16, and 32. At the baseline, 22% of individuals with newly acquired HIV are diagnosed during the early infection stage. As the diagnosis rates increase incrementally, the proportions of diagnosed individuals rise to 39%, 62%, 86%, 98% and 100%, respectively.
  - **Accelerated diagnosis in individuals who acquired HIV within the previous 6 months.** This scenario captures the effect of an intervention that causes an increase in diagnosis rates not only in individuals with early HIV infection as in (b) but also in individuals who have entered the chronic HIV infection but have acquired HIV within the previous 6 months. Similarly, the diagnosis rate is increased (i.e., 2, 4, 8, 16, and 32-fold) compared to the baseline diagnosis rate, and individuals initiate ART on the same day. At the baseline, 36% of individuals who acquired HIV are diagnosed during the first 6 months of infection. As the diagnosis rates increase incrementally, the proportions of diagnosed individuals rise to 60%, 84%, 97%, 99.99%, and 100%, respectively.

### Model outcomes

To evaluate the intervention impact, we considered the annual numbers of new HIV infections and new HIV diagnoses, the cumulative number of new HIV infections averted and reduction in HIV diagnoses, as well as the distribution of the annual number of new HIV diagnoses by the duration between HIV acquisition and diagnosis. To facilitate comparison between the data and model outputs, we report the latter by adapting the classification used by SHM in the annual reports. This classification stratifies stage of HIV infection at diagnosis as 0-6 months, 6-12 months, more than 12 months. The cumulative number of new HIV infections averted and reduction in HIV diagnoses were calculated over 10 years starting from the intervention implementation on 1 January 2023. For each scenario, we generated 20 trajectories for each of 100 parameter sets obtained through the calibration process. We summarized the distribution of the model outputs by calculating median of measurements over 20 trajectories for each parameter set and subsequently presenting the outputs as medians and 95-th quantile interval (95-th QI) of measurements over 100 parameter sets.

### Sensitivity analyses

We investigated the contribution of immediate initiation of ART on the impact of the intervention on HIV transmission dynamics. We also have considered an impact of the intervention which may increase diagnosis rates in individuals whose history of HIV infection exceeds 6 months but is shorter than one year. The full description of the sensitivity analyses is provided in the Subsections Sensitivity to parameter perturbation and Immediate initiation of ART in Section Additional Analyses in S2 Appendix.

## Results

### Model fit

**Fig 1:**
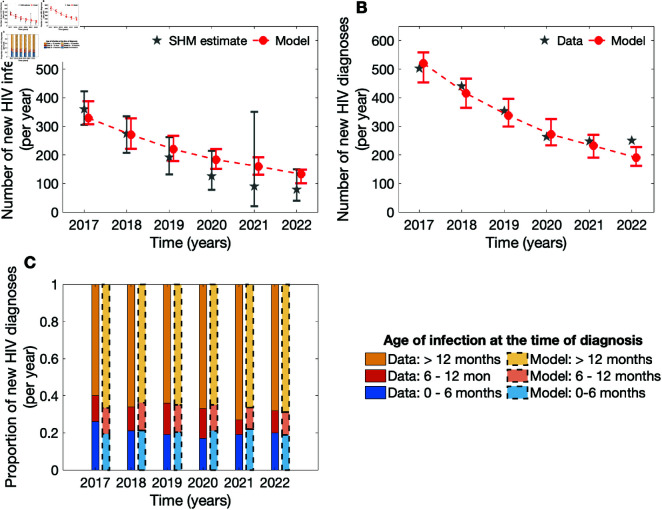
Model fit to the HIV surveillance data and SHM-provided estimates among MSM in the Netherlands. a Newly acquired HIV infections, b newly diagnosed HIV infections, and c distribution of the number of new HIV diagnoses by the duration between HIV acquisition and diagnosis for MSM, as estimated in the model and reported by SHM in the Netherlands. In a and b, the red dots connected by the dashed line depict the baseline model output. For the data, the large grey stars and the bars denote the median estimates and the confidence intervals, respectively. For the model outputs, the large red dots and the bars denote the median estimates and the 95-th QI, respectively. In c, the bar chart colors correspond to the time between HIV acquisition and diagnosis. For each year, bars with saturated colors and solid borders on the left correspond to the data, while the bars with less saturated colors and dashed borders on the right correspond to the model output.

The model fit to key data statistics of interest is shown in Fig 1. There was strong agreement between the model outputs and the data, namely the SHM estimate of the annual numbers of new HIV infections (Fig 1a) and new HIV diagnoses (Fig 1b). The model estimates of the proportions of individuals who received an HIV diagnosis within the first 6 and between 6 and 12 months following HIV acquisition marginally exceeded the data estimates (Fig 1c).

### Maximum impact of the intervention

**Fig 2:**
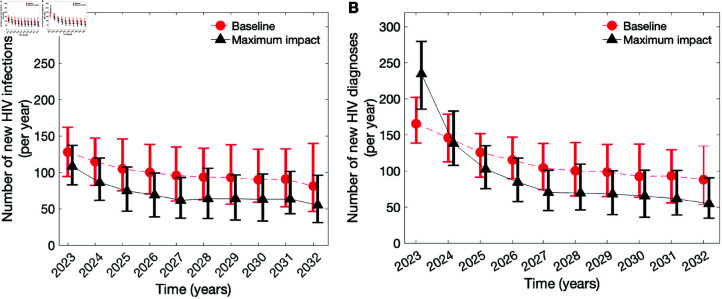
Model predictions for the maximum impact intervention. (a) Newly acquired HIV infections and (b) newly diagnosed HIV infections in the baseline and maximum impact model scenarios. The red dots connected by the dashed line depicts the baseline scenario without additional diagnosis of individuals with early infection. The black triangles connected by a solid line depict the maximum expected impact of an intervention where individuals with early infection are diagnosed immediately after HIV acquisition and start ART immediately at the time point of receiving the diagnosis. The dynamics are summarized by the median estimates (circles and triangles) and the IQ (bars).

**Fig 3:**
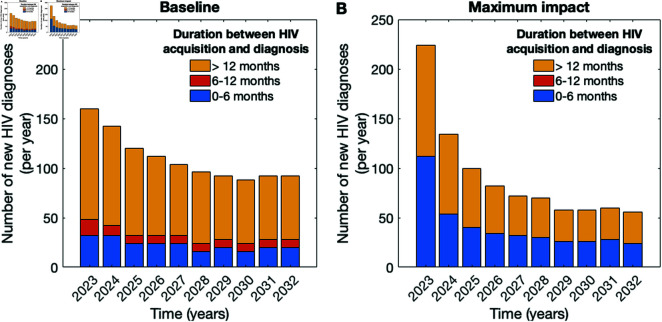
Distribution of new HIV diagnoses by the duration between HIV acquisition and diagnosis. (a) The baseline scenario without an increase in the diagnosis rate of individuals with early infection. (b) The maximum impact intervention combines immediate diagnosis following acquisition of HIV combined with immediate ART initiation. Bar chart colors correspond to the duration between HIV acquisition and the time of diagnosis.

The comparison of the baseline model scenario with the maximum impact scenario is shown in Figs 2 and 3. Specifically, we examined the annual numbers of new HIV infections (Fig 2a) and new HIV diagnoses (Fig 2b). Furthermore, we assessed the distribution of the annual number of new HIV diagnoses by the duration between HIV acquisition and diagnosis to evaluate the intervention’s time frame of efficiency (Fig 3). **New HIV infections.** Our analyses indicated that in the context of continually decreasing low annual numbers of new HIV infections, as observed among MSM in the Netherlands, an intervention facilitating increased diagnosis of individuals with early HIV infection combined with immediate initiation of ART can further expedite this downward trend (Fig 2a). The effects of such intervention can become noticeable as early as the first year after implementation. In the baseline scenario, we anticipate the median annual number of new HIV infections to decline from 128 (95-th QI: 94–161) in 2023 to 81 (95-th QI: 46–138) in 2032. In the maximum impact scenario, we expect the median annual number of new HIV infections to decrease from 108 (95-th QI: 83–137) in 2023 to 55 (95-th QI: 31–96) in 2032, resulting in a cumulative decrease of 298 (95-th QI: 162–451) over 10 years. Despite the fact that all individuals who newly acquire HIV are diagnosed almost immediately, onward transmission may still occur in the model due to undiagnosed individuals with chronic HIV infection remaining undiagnosed and undiagnosed HIV infections imported from abroad. Furthermore, individuals who experience ART failure or drop out from ART programme may transmit HIV until the time when they achieve a suppressed viral load again.

**New HIV diagnoses.** The analysis of the annual number of new diagnoses (Fig 2b) indicated that, in the maximum impact scenario, there will be a sharp increase in the median number of newly diagnosed HIV infections from 166 (IQR: 139–202) at baseline to 235 (186–280) in the year following the start of the intervention. However, the number of HIV diagnoses is expected to decrease to the baseline scenario level within the first two years of the intervention onset and subsequently drop significantly below it by the third year. This drastic decrease in newly diagnosed HIV infections signifies an overall reduction in HIV transmission induced by the intervention. In the maximum impact scenario, the model predicted a decrease in the cumulative number of diagnoses of 182 (95-th QI: 77–315) over 10 years.

**Distribution of new HIV diagnoses by the duration between HIV acquisition and diagnosis.** To analyze the window of efficiency of the maximum impact intervention, we examined the distribution of new HIV diagnoses by the duration between HIV acquisition and the time of diagnosis (Fig 3). In the baseline scenario, our model predicted that the majority of individuals continue to be diagnosed more than 12 months after HIV acquisition (Fig 3a). For the maximum impact intervention, the number and proportion of diagnoses in individuals with early HIV infection increased drastically in the first three years of the intervention (Fig 3b) and continued the exceed baseline levels in the subsequent years. Specifically, over the first three years, in the baseline scenario, a median of 80 (95-th QI: 44–132) out of 422 (95-th QI: 296-564) new diagnoses were in individuals who acquired HIV within the previous 6 months. In the maximum impact scenario, a median of 206 (95-th QI: 128–292) out of 458 (95-th QI: 320–616) individuals were expected to receive a diagnosis within the first 6 months following HIV acquisition. In both baseline and maximum impact scenarios, the number of new diagnoses in individuals infected in the first 6 months following HIV acquisition stabilizes towards the end of the time horizon with median 20 (95-th QI: 8–36) and 24 (95-th QI: 16–60), respectively.

### Attaining maximum impact

**Fig 4:**
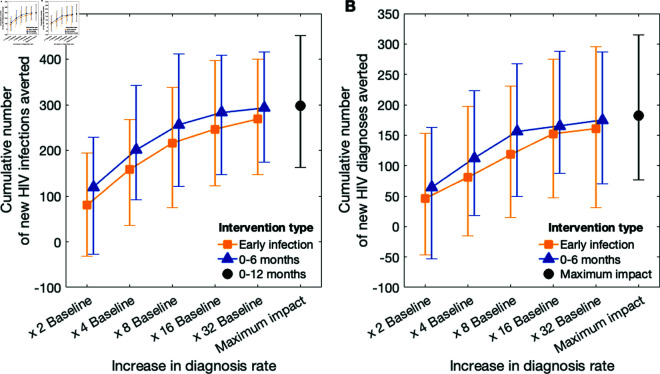
Impact of the intervention across different scenarios. (a) Cumulative number averted of HIV infections and (b) cumulative HIV diagnoses over 10 years. Yellow squares and blue triangles correspond to the intervention where the diagnosis rate is increased, compared to the baseline, in individuals with early HIV infection or those who acquired HIV within the previous 6 months, respectively. The black dots mark the maximum impact scenario.

To identify the increase that results in a reduction (relative to the baseline) of the number of new HIV infections comparable to that of the maximum impact intervention we investigated a range of diagnosis rates for individuals with early HIV infection (Fig 4). Additionally, we considered the impact of an extended intervention, where the diagnosis rate is increased in all individuals who acquired HIV infection within the previous 6 months. To perform these analyses, we calculated the cumulative number of averted HIV infections (Fig 4a) and diagnoses (Fig 4b) over 10 years.

**Accelerated diagnosis in individuals infected with early HIV infection.** A 2-fold increase in the diagnosis rate of individuals with early HIV infection led to 80 (95-th QI: -32–195) total infections averted in 10 years, with 31 (95-th QI -234–334) of which were averted in the first five years (Fig 4a). An 8-fold increase in the diagnosis rate of early infections reduced the median cumulative infections to 216 (95-th QI: 75–338). The 8-fold increase had a more immediate impact than the 2-fold increase, with 95 (95-th QI: -155–417) infections averted in the first five years. A 16-fold increase resulted in 247 (95-th QI: 122–398) total infections averted, 114 (95-th QI: -123–450) of which occurred in the first five years. Finally, a 32-fold increase in the diagnosis rate resulted in 269 (95-th QI: 148-400) infections averted. However, this outcome is not proximate to the maximum impact of the intervention [i.e., 298 (95-th QI: 162–451) infections averted]. Notably, in the first five years, the number of infections averted in the 32-increase intervention was almost equal to the 16-fold increase intervention [i.e., 114 (95-th QI: -123–450) vs. 128 (95-th QI: -100–435) cumulative infections, respectively]. The trends we observed in the decrease in the cumulative number of new HIV infections were respectively reflected in the cumulative number of new HIV diagnoses (Fig 4b). Thus, an intervention that accelerates diagnosis in individuals with early HIV infection should aim for a sufficiently high increase in the diagnosis rate. However, once this threshold is reached, no additional benefit can be attained through further increases. Instead, consideration should be given to an additional expansion of the intervention involving a larger group of MSM with undiagnosed HIV infection.

**Accelerated diagnosis in individuals infected within the previous 6 months.** To identify such expansion, we investigated the outcomes of an intervention with an increased diagnosis rate in individuals who acquired HIV within the previous 6 months. We observed that this intervention yields a larger number of infections averted (Fig 4a, blue triangles) throughout the range of increases in the diagnosis rate that we considered. For a 16-fold or larger increase in the diagnosis rate, in individuals who acquired HIV within the previous 6 months predicted to result in 282 (95-th QI: 147–408) infections averted. Also, expansion of the intervention to individuals who acquired HIV infection within the last 6 months requires a lower increase in diagnosis rate, i.e. 8 times, yields comparable outcomes to the intervention that captures only individuals with AEHI but requires an increase of 32 times, with 256 (95-th QI: 122–411) and 269 (95-th QI: 147–400) infections averted. Finally, the increase of the diagnosis rate by a factor of 8, in the intervention with scope extending to individuals who acquired HIV in the last 6 months results in the largest absolute decrease in the number of diagnoses (median of 37).

For the full tabulation of scenarios’ outcomes as presented on Fig 4 please refer to Section Complete statement of the results for incremental increases in diagnosis rate in Supplementary Materials 1.

### Sensitivity analyses

We explored the contribution of ART immediately after receiving an HIV diagnosis on the impact of the intervention. Repeating the analysis from the previous section, we examined the 10-year trends in both new HIV infections and diagnoses across various diagnosis rates among individuals with early HIV infection and those infected in the last 6 months. This time, individuals diagnosed through the intervention were assumed to start ART at the same rate as those identified via routine testing. Our findings suggest that, in our study context, under conditions similar to the contemporary Netherlands, when the increase in diagnosis rates is small, the added benefit of immediate ART initiation on reducing HIV incidence is not substantial beyond the impact of accelerated diagnosis. However, with larger increases in diagnosis rates, the immediate initiation of ART can result in averting a significant number of new HIV infections. However, even for large increases in the diagnosis rate, the contribution of ART is significantly smaller than that of the accelerated diagnosis.

Finally, we investigated whether expanding the scope of the intervention to include individuals who acquired HIV within the last 12 months would yield further benefits. We assumed an equal diagnosis rate for individuals infected between 6 and 12 months prior and those infected within the previous 6 months. Our projections show that this scenario could result in further reductions in the number of HIV infections and diagnoses. The most substantial improvement relative to the 6-months intervention is expected for a smaller increases in the diagnosis rate (2- and 4-fold), with diminishing returns for larger diagnosis rates. In contrast, when considering reduction in the cumulative number of HIV diagnoses, a progressive increase in the diagnosis rate, results in comparable gains across different rates. It is important to note that while the increase in diagnosis rates is multiplicative, the cumulative reduction in the number of HIV diagnoses is additive.

The detailed analyses are provided in the Subsections Immediate initiation of ART and Increase in the scope of the intervention in Section Additional Analyses in S2 Appendix.

## Discussion

In this study, we developed an agent-based model to investigate the potential impact of an intervention accelerating the diagnosis of MSM with early HIV infection, combined with immediate ART initiation, on the HIV epidemic among MSM in the Netherlands. Our analysis revealed that the intervention could significantly contribute to the decline in new HIV infections, particularly when diagnosis rates for early infection are thoroughly optimized. The model suggests that for individuals with early HIV infection a 32-fold increase in the diagnosis rate, corresponding to 100% of new HIV infections diagnosed during early HIV infection, is necessary to approach the maximum impact of the intervention. The size of the increase was determined through modeling and was not derived from an empirical study. We note that achieving and maintaining such a high increase may not be feasible or cost-effective. Therefore, we considered other implementation strategies to maximize the intervention’s impact. By expanding the intervention to include individuals who have acquired HIV within the previous 6 months, an 8-fold increase in the diagnosis rate was shown to yield a similar impact to that of a 32-fold increase when targeting only individuals with early HIV infection. Importantly, while our model focused on increasing diagnosis rates combined with immediate ART for HIV infections acquired within the previous 6 months only, the real-world implementation of this intervention would likely also identify individuals with HIV infection older than 6 months as well. These individuals, upon being diagnosed through the intervention, would not be excluded from immediately receiving ART. This approach would result in a greater impact, as it captures and offers treatment to a broader spectrum of the HIV-infected population, not just those within the modeled 0-6 month period. Our sensitivity analyses confirm this assumption, especially when the increase in the diagnosis rate is relatively small.

Our model aimed to accurately capture HIV transmission dynamics among MSM by portraying a realistic depiction of the dynamics of the sexual network among MSM. This portrayal was informed by data on partnership formation patterns and duration among MSM in the Netherlands. Key aspects relevant to HIV transmission, including serosorting, age assortativity, and condom use differentiated by partnership type and participant age, were integrated. We also modeled the standard of HIV care and prevention in the Netherlands. Furthermore, over the last 8 years, the annual incidence of new HIV diagnoses was declining and as of 2022 is relatively low, signaling the decline in the incidence of new HIV infections within the Netherlands due to local dynamics. However, this trend suggests that importation of infections from abroad may affect the local epidemic and impact the HIV elimination scenarios. Therefore, the model accounts for the importation of HIV infections, arising either through travel of Dutch residents or through immigration. Finally, the model’s outputs were calibrated against relevant epidemiological statistics, specifically the incidence of new HIV diagnoses and the stage of infection at the time of HIV diagnosis, lending credibility to its projections and enhancing the trustworthiness of our findings.

The importance of early HIV infection in the context of HIV elimination has received attention in modeling literature for well over a decade [32] with studies demonstrating that the accelerated diagnosis of early HIV infection can be a successful strategy for reducing transmission among MSM. For example, a study by Dimitrov et al. [33] for MSM and transgender women set in Peru, a country with a notably HIV incidence, projected that a significant number of new HIV infections could be prevented over 20 years by increasing the diagnosis rate and initiating ART early in individuals with early HIV infection. We expect that the differences in the magnitude of projections between this study and ours can be explained by the state of the care cascade at the start of the intervention. Dimitrov et al. [33] estimated that in 2018, only 19% of all MSM and transgender women living with HIV were virally suppressed, while our study, using SHM data, indicated this metric to be around 90% among MSM [1]. The study by Gurski et al. [34] had shown similar findings, highlighting the potential benefits of accelerating diagnosis in individuals with early HIV, in a modeling study for the general MSM population in the USA. In the study, the increase in early HIV diagnosis and ART uptake led to notable reductions in the number of new HIV infections and a subsequent decrease in HIV prevalence over a two-decade span. The importance of immediate ART initiation varied across studies. For example, a study focusing on MSM in San Francisco underscored the importance of prompt ART initiation, showing that reducing the time from diagnosis to treatment initiation could significantly decrease the number of new HIV infections [35]. In contrast, a modeling study by Kusejko et al. [36] in Switzerland found that early initiation of ART resulted in only a modest decrease in the expected number of new HIV infections as compared to increasing HIV testing rates among individuals with early HIV infection. In our study for MSM in the Netherlands, we arrived at a similar conclusion to that of Kusejko et al. [36]. This suggests that the effectiveness of accelerated ART initiation in reducing onward HIV transmission may depend on the epidemic context. This latter can depend on other interventions rolled out in the population. To wit, in the Netherlands, in 2019 a 5-year long PrEP programme with capacity of 8,500 was rolled out, contributing to decrease of incidence of new HIV infections. Analysis by Reitsema et al. [37] has shown that if the programme continues and its capacity is expanded to 16,000 participants, additional reduction in incidence of HIV infections can be achieved. In this context, the impact of the accelerated diagnosis of individuals with early HIV infection may be lower than what our study is projecting.

Our model has several limitations, including the omission of the eclipse phase, the time between HIV acquisition and the first appearance of detectable viral RNA in plasma [38], and the simplification of care retention and prevention program progression. The impact of external factors, such as the COVID-19 pandemic, on sexual risk behavior also remains unaccounted for. Not accounting for these factors can affect the numerical accuracy of the models’ predictions by making the intervention appear more or less effective than it realistically can be expected under these conditions. For instance, not accounting for the eclipse stage when viral RNA is undetectable can lead to overestimation of the ability to diagnose individuals with early HIV infection. On the other hand, simplifying the details of care retention might lead to optimistic projections regarding the long-term engagement in the care of diagnosed individuals including those who were diagnosed via the early diagnosis intervention. These limitations highlight the importance of incorporating more detailed data on the eclipse phase and the nuances of care retention and prevention program progression for more accurate and actionable projections. While we acknowledge that not all MSM engage in AI by age 15 or at all, our model does not differentiate these behaviors, instead assuming that, under similar conditions, a uniform rate of AI among all individuals based on averaged data applies. This approach simplifies the modeling process, and while it may not capture the diverse sexual behaviors in the MSM population, it is designed to maintain an accurate average contact rate for the population studied. We have not modeled loss to follow up in individuals who were not retained in care following the diagnosis. While ensuring continuous care for these individuals is important for eliminating the HIV epidemic, they are not the focus of the intervention considered here. It is important to note that retention in care is very high among MSM in the Netherlands, with 97% of those known to be diagnosed and alive still engaged in care as of 2022 [1]. Finally, we acknowledge that starting the simulation relatively recently, in 2016, rather than from the onset of the HIV epidemic in the 1980s introduces some uncertainty into the model. While our approach arose from the availability of relevant data, it can affect the robustness of the model’s initial conditions and parameter estimates. On the other hand, fitting the model to reflect several decades of HIV epidemic would require implementing changes in both HIV prevention and treatment policies, and behavioral changes as a response to these policies. Surveillance and detailed sexual behavior data are not consistently available for the entire period, making it unlikely that fitting the model from the start of the epidemic would lead to more robust parameter estimates. Together these factors might introduce some degree of uncertainty to the precise quantification of the intervention’s impact, but it is essential to note that our model accurately reproduces key epidemiological statistics central to our analysis, both numerically and in terms of dynamical trends. Consequently, we are confident that the core insights and directional trends predicted by our model will remain valid in similar HIV transmission contexts, as pertains to the low decreasing number of new HIV infections and a cascade of care proximate to UNAIDS’ 95-95-95 targets.

Inspired by targeted screening strategy for early HIV infection and immediate ART initiation among MSM at the Amsterdam sexually transmitted infection clinic [9], which focused on increasing early infection diagnosis through awareness campaigns combined with a robust HIV-risk score, same-day diagnosis, and immediate ART initiation, our study demonstrates the potential for expanding such strategies on a national scale. However, their success depends on the local epidemiological and social landscapes, underscoring the need for context-specific adaptations.

Mathematical modeling can elucidate how to adjust these interventions for varying contexts to optimize their impact, capturing diverse epidemiological profiles and sexual behaviors to tailor public health strategies. To effectively implement the interventions that our model explored, a comprehensive needs assessment will be essential. This assessment can identify barriers to engaging with interventions, such as access to information, the ability to undergo screening and HIV testing promptly, and the initiation and adherence to ART treatment. Factors including place of residence, age, and socio-demographic status significantly influence these aspects [39]. Our model primarily aimed at assessing the potential impact of increased diagnosis rates on reducing HIV transmission among MSM. As such, it directly focused on the increase in diagnosis rates without capturing the corresponding increase in screening and testing that such an intervention requires. Therefore, we did not evaluate the practical feasibility of implementing the scenarios considered in this work, such as the resources and infrastructure needed to achieve the modeled increases in diagnosis rates. Understanding the full scope of the intervention’s impact requires examining the intricacies of testing and screening processes, as well as considering the challenges in engaging the target population to achieve a necessary increase in diagnosis rates. Finally, our research presented in this manuscript focused specifically on accelerated diagnosis of early HIV. To determine whether accelerated diagnosis of early HIV combined with immediate ART initiation could be a feasiable public health intervention, future research should compare its impact with other strategies, such as increasing PrEP coverage, and include a cost-effectiveness evaluation.

The success of targeted screening and immediate ART initiation in Amsterdam, supported by media campaigns developed with the MSM community, underscores the potential of such approaches [10]. Yet, the effectiveness of replicating these strategies in regional contexts different from Amsterdam highlights the complexity of adapting interventions to different cultural landscapes. For instance, the original campaign’s impact in Amsterdam facilitated through collaboration with community stakeholders and tailored media strategies, may not directly translate to regions with distinct cultural and socio-demographic characteristics [3]. Differences in cultural acceptance, population density, and accessibility to campaign materials and testing and care services could markedly affect intervention outcomes. Future work should focus on tailoring interventions to accommodate regional variations in HIV dynamics and barriers to screening and early diagnosis, ensuring more universally effective HIV prevention strategies.

Our analyses did not explore the implications of health behavior heterogeneity related to immigration or mobility of MSM. However, according to HIV surveillance data, the rate of new HIV infections originating within the Netherlands is decreasing. It is conceivable that within 10 years, the importation of new HIV infections may become a driving force in HIV transmission dynamics. This trend is already gaining attention in Denmark as demonstrated by a study by Palk et al. [40]. Consequently, the intervention aiming to increase the diagnosis of individuals with early infection may need to change its approach in order to ensure engagement of targeted population with media intervention as well as the ability to act on the information received. The latter could be influenced by factors such as perceived stigma, trust in, and access to healthcare [41]. This situation calls for the development of new, more tailored interventions aimed at addressing these barriers.

## Conclusion

Our study demonstrates that accelerating early-stage HIV diagnosis can significantly lower new infections among MSM, particularly in settings like the modern-day Netherlands, where UNAIDS’ 95-95-95 targets are met. Key to translating these findings into practice is tailoring strategies to the unique needs and conditions of the local population. Our modeling, which showed the impact of increasing diagnosis rates to desired levels, assumed high retention in care and optimal ART adherence. To achieve the impact that our study projected, essential factors including engaging MSM effectively, ensuring access to testing and immediate ART initiation, and supporting sustained ART adherence need to be in place. By conducting thorough needs assessments and engaging with stakeholders, we can devise targeted early diagnosis strategies, moving closer to eliminating HIV transmission.

## Supporting information

S1 Appendix Model structure and parametrization. Full description of the model structure and parametrization.
(PDF)

S2 Appendix Additional analyses Additional analyses supplementing results presented in the main text and sensitivity analyses of the findings to immediate initiation of ART.
(PDF)

## Acknowledgments

We thank Peter Reiss, Wim Zuilhof, Marleen Werkman, Martin Bootsma, Michiel van Boven, Kim Romijnders, Maarten Reitsema, Maria Xiridou, Don Klinkenberg, and the Dutch HIV Association for useful discussions.
