# Supplementary material for: Impact of increased diagnosis of early HIV infection and immediate antiretroviral treatment initiation on HIV transmission among men who have sex with men in the Netherlands
Source: PLoS Comput Biol. 2025 Feb 27;21(2):e1012055. doi: 10.1371/journal.pcbi.1012055 (PMC11882050; doi:10.1371/journal.pcbi.1012055)
Supplement: S2 Appendix — Additional analyses supplementing results presented in the main text and sensitivity analyses of the findings to immediate initiation of ART. (PDF) [file pcbi.1012055.s002.pdf]

Supplementary Information: Impact of increased diagnosis of early HIV infection and immediate antiretroviral treatment initiation on HIV transmission among men who have sex with men in the Netherlands

Alexandra Teslya[1*], Janneke Cornelia Maria Heijne[2,3,4,5], Maarten Franciscus Schim van der Loeff[2,5,6], Ard van Sighem[7], Jacob Aiden Roberts[1], Maartje Dijkstra[5], Godelieve J. de Bree[5], Axel Jeremias Schmidt[8,9], Kai J. Jonas[10], Mirjam E. Kretzschmar[1,11,12], and Ganna Rozhnova[1,11,13,14]

[1]Julius Center for Health Sciences and Primary Care, University Medical Center Utrecht, Utrecht University, Utrecht, The Netherlands
[2]Department of Infectious Diseases, Public Health Service of Amsterdam, Amsterdam, The Netherlands
[3]Amsterdam institute for Immunology & Infectious Diseases (AII), Amsterdam UMC, University of Amsterdam, Amsterdam, The Netherlands
[4]Amsterdam Public Health research institute (APH), Amsterdam UMC, University of Amsterdam, Amsterdam, The Netherlands ,
[5]Department of Internal Medicine, Division of Infectious Diseases, Amsterdam Institute for Immunology and Infectious Diseases, Amsterdam UMC, University of Amsterdam, Amsterdam, The Netherlands
[6]Amsterdam UMC location University of Amsterdam, Amsterdam Public Health Research Institute, Amsterdam, The Netherlands
[7]Stichting HIV Monitoring, Amsterdam, The Netherlands
[8]Sigma Research, Department of Public Health, Environments and Society, London School of Hygiene and Tropical Medicine, London, United Kingdom
[9]Medicine and Health Policy Unit, German AIDS Federation, Berlin, Germany
[10]Faculty of Psychology and Neuroscience, Maastricht University, Maastricht, The Netherlands
[11]Center for Complex Systems Studies (CCSS), Utrecht University, Utrecht, The Netherlands
[12]Institute of Epidemiology and Social Medicine, University of Münster, Münster, Germany
[13]BioISI – Biosystems & Integrative Sciences Institute, Faculdade de Ciências, Universidade de Lisboa, Lisbon, Portugal
[14]Faculdade de Ciências, Universidade de Lisboa, Lisbon, Portugal
[*]Corresponding author, e-mail: a.i.teslya@umcutrecht.nl

Complete statement of the results for incremental increases in diagnosis rate

In this section we provide full description of results shown on Fig 4, in Subsection Attaining maximum impact, Section Results in the main manuscript (Table A).

S2 Table A: **Cumulative number of HIV infections averted and cumulative reduction in diagnoses over 10 years**. Columns with title AEHI refer to interventions with scope of effect in AEHI individuals only, while title 0-6 months refers to the intervention where the scope of effect extended all individuals who were infected within previous 6 months.

	**HIV infections Median (95-th QI)**		**HIV diagnoses Median (95-th QI)**	
Intervention increase	AEHI	0-6 months	AEHI	0-6 months
×2 Baseline	80 (-32–195)	120 (-28–228)	46 (-46–153)	64 (-53–163)
×4 Baseline	158 (35–267)	201 (92–343)	80 (-15–198)	112 (18–223)
×8 Baseline	216 (75–338)	256 (122–411)	118 (15–231)	156 (49–267)
×16 Baseline	247 (122–398)	283 (147–408)	152 (47–275)	165 (87–288)
×32 Baseline	169 (147–400)	293 (174–415)	161 (31-295)	175 (69-187)
Maximum	298 (162–451)		183 (77–315)	

Sensitivity analyses

Immediate initiation of ART

In this section, we provide a summary of the exploration of the effect of initiation of ART on the same day when HIV diagnosis is received on the overall impact of the intervention. To achieve this, we repeated the simulations described in Subsection *Attaining maximum impact*, where we varied the diagnosis rate for individuals who were either in the early stage of HIV infection or who acquired HIV infection within the previous 6 months. In these experiments, individuals who were diagnosed through the intervention route started ART at the same rate as the rest of the population. The results are shown in S2 Fig A. We observed that in conditions similar to the modern Netherlands, if the increase in the diagnosis is on the smaller end of the interval, the ART initiation does not have a significant effect on further reducing HIV incidence, beyond the effects of accelerated diagnosis. In all scenarios which we explored, the difference in terms of cumulative number of HIV infections averted fluctuated between 2 and 10, which we deem not statistically significant, given the scaling factor of between from the model population size to the real population size.

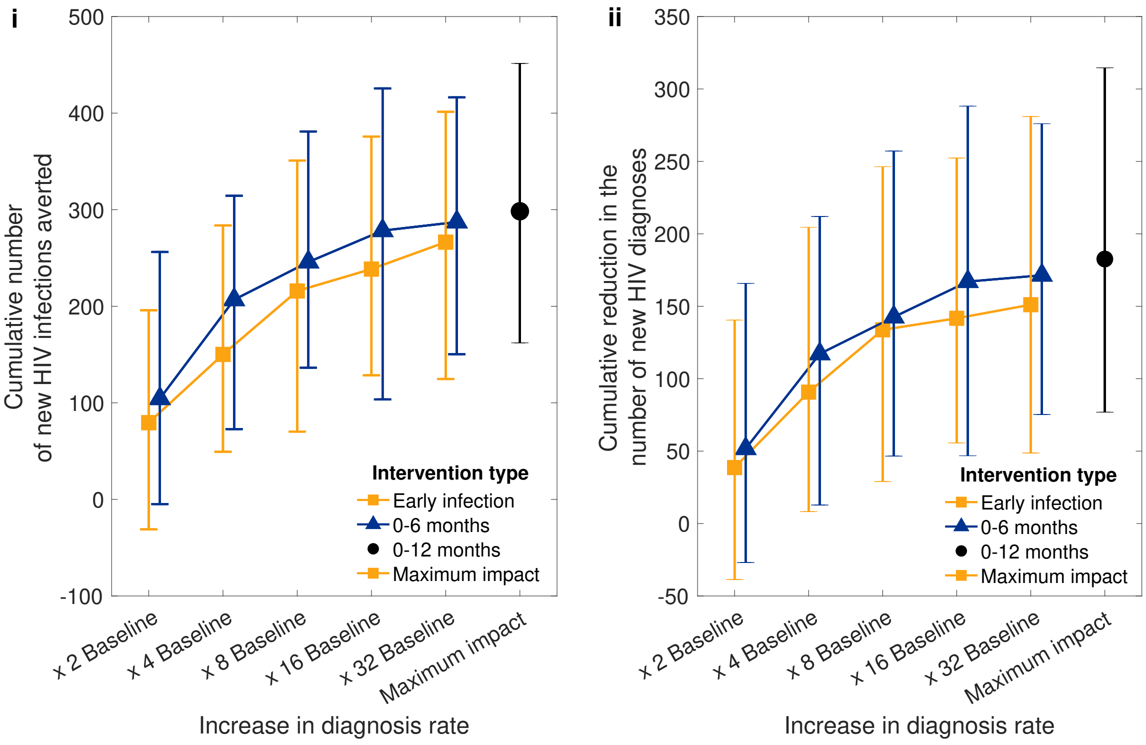

S2 Fig A: **Impact of the intervention across different scenarios without immediate initiation of ART for individuals diagnosed via participation in the intervention.** (**i**) Cumulative HIV infections and (**ii**) cumulative HIV diagnoses over 10 years. Red diamonds correspond to the baseline scenario without an additional increase in the diagnosis rates. Yellow dots and blue triangles correspond to the intervention where the diagnosis rate is increased, compared to the baseline, in individuals with early HIV infection or those who acquired HIV within the previous 6 months, respectively.

Extending the scope of the intervention to individuals who acquired infection in the previous 12 months

In this subsection, we consider the effect of the intervention in the scenario where the diagnosis rates of individuals who acquired the infection in the previous 12 months increase. We implement this scenario by setting the diagnosis rate for individuals with chronic HIV infection who acquired 6-12 month previously to the same value as the diagnosis rate in individuals who acquired HIV within the previous 6 months (S2 Fig B). This scenario is optimistic to what can possibly be achieved in real life, since individuals with chronic infection experience very little symptoms and the re-collection of potential exposure event may be less recent, resulting in lower testing rate and therefore, lower diagnosis rate. It should be noted that the effectual increase in diagnosis rate for individuals who were infected more than 6 months ago is higher than what is indicated by the tick. We investigate this scenario using the cumulative number of HIV infections and the cumulative diagnoses over a 10-year period

In this scenario, we observe that if the intervention manages to reach the increase in diagnosis rate in individuals who acquired HIV infection more than 6 months previously, there are additional decreases in terms of the number of new HIV infections and diagnoses. Lower increases in the diagnosis rate (factors of 2 and 4) result in higher gains in terms of averted HIV infections. (S2 Fig B**i**). In contrast, when considering the cumulative reduction in the number of new HIV diagnoses we see that the gains increase linearly throughout the range of diagnosis rate increases (S2 Fig B**ii**).

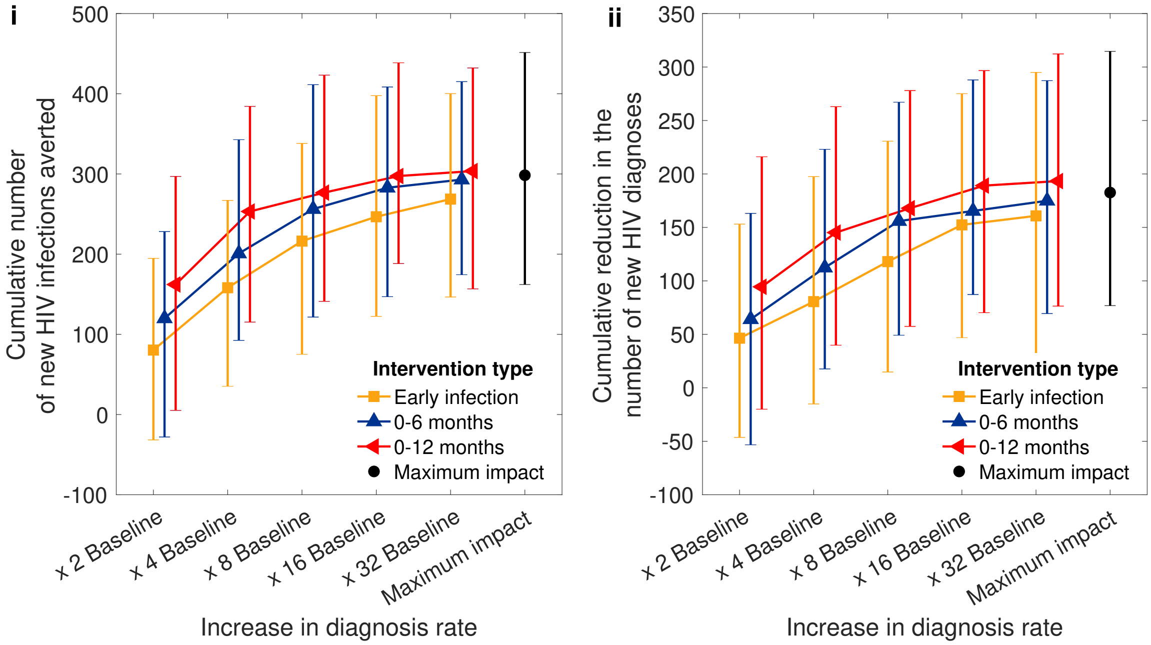

S2 Fig B: **Impact of the intervention across different scenarios.** (**i**) Cumulative HIV infections and (**ii**) cumulative HIV diagnoses over 10 years. Yellow squares, blue upward-pointing triangles, and red left-pointing triangles correspond to the intervention where the diagnosis rate is increased, compared to the baseline, in individuals with early HIV infection or those who acquired HIV within the previous 6 and 12 months, respectively.

Complimentary data for the results presented in the main text

Distribution of the age of infection at the time of diagnosis

In Table B we compare distribution of age of infection at the time of diagnosis in surveillance data to the distribution simulated by the model during the calibration period.

References

S2 Table B: Distribution of stage of HIV infection at the time of diagnosis.

Year		0-6 months M (95 IQR)†	6-12 months (M, 95 IQR)	Later than 12 months (M, 95 IQR)
2017	Model	0.22 (0.18-0.26)	0.13 (0.10-0.17)	0.65 (0.58-0.68)
	Data	0.26	0.14	0.6
2018	Model	0.22 (0.18-0.28)	0.13 (0.09-0.17)	0.65 (0.59-0.71)
	Data	0.21	0.13	0.66
2019	Model	0.21 (0.17-0.29)	0.13 (0.10-0.16)	0.66 (0.58-0.71)
	Data	0.19	0.17	0.64
2020	Model	0.21 (0.27-0.71)	0.12 (0.09-0.16)	0.67 (0.61-0.72)
	Data	0.17	0.16	0.67
2021	Model	0.20 (0.15-0.26)	0.12 (0.08-0.15)	0.66 (0.60-0.72)
	Data	0.19	0.0.08	0.73
2022	Model	0.20 (0.14-0.28)	0.11 (0.08-0.15)	0.70 (0.61-0.74)
	Data	0.2	0.12	0.68

† M stands for the median, and 95 IQR refers to the interquantile range (2.5th-97.5th percentiles). Median and IQR apply only to model outputs.
